# Synthetic Nicotine Analogue, Nicotine and CBD in US-marketed “Tobacco-Free” Cigarettes

**DOI:** 10.21203/rs.3.rs-11528001/v1

**Published:** 2026-10-29

**Authors:** Sairam V. Jabba, Ana Caceres, Peter Silinski, Zhong Li, Sven Jordt

**Affiliations:** Duke University School of Medicine; Duke University School of Medicine; Duke University; Duke University School of Medicine; Duke University School of Medicine

**Keywords:** Tobacco, cigarettes, nicotine, nicotine analogues, 6-methyl nicotine, CBD, cannabidiol, cannabinoids, hemp, addiction, menthol

## Abstract

**Background and aims:**

Electronic cigarette and oral pouch products containing the nicotine analogue 6-methyl nicotine have become available in many parts of the world, evading tobacco and nicotine regulations. Recently, we identified United States-marketed “tobacco-free” combustible cigarette products that may contain 6-methyl nicotine, available in “regular” and “menthol” flavours. The present study aimed to verify the presence of 6-methyl nicotine and menthol and the absence of nicotine in “tobacco-free” cigarettes, and to identify the source of the plant materials used in manufacturing.

**Methods:**

Choice^™^ “tobacco-free” cigarettes and control tobacco cigarettes were purchased in physical locations and online stores in the United States. Cigarette rods and papers were extracted and analyzed by gas chromatography-mass spectrometry (GC-MS) and ultra performance liquid chromatography-mass spectrometry (UPLC-MS).

**Results:**

6-methyl nicotine was identified in Choice^™^-cigarettes, at similar quantities in both flavour varieties (regular: 455 ± 59 μg/rod vs. menthol: 484 ± 36 μg/rod) and across purchase sources. Newport regular and menthol tobacco cigarettes contained nicotine at 9119 ± 633 and 7050 ± 721 μg/rod. Nicotine was also detected in both Choice^™^-cigarette flavour varieties (7.4 ± 1.3 μg/rod). Menthol contents in Choice^™^-Menthol cigarettes ranged between 2472 ± 297 μg/rod and 3362 ± 510 μg/rod, higher than in Newport menthol tobacco cigarettes (2222 ± 13 μg/rod). Cannabidiol (CBD) was identified in both flavor varieties of Choice^™^ cigarettes (regular: 9271 ± 1024 μg/rod; menthol cigarettes: 11292 ± 1629 μg/rod) as well as smaller quantities of other cannabinoids.

**Conclusions:**

We report the emergence of “tobacco-free” combustible cigarette products containing a synthetic nicotine analogue, 6-methyl nicotine. While the determined 6-methyl nicotine contents are lower than nicotine contents in tobacco cigarettes, 6-methyl nicotine’s higher potency may elicit similar reinforcing effects. The presence of substantial amounts of CBD, along with other cannabinoids, suggests hemp as the filler plant material.

Legislators and regulators need to urgently address combustible nicotine analogue products that threaten to undermine smoking prohibitions, taxation, age limitations and flavour regulations.

## INTRODUCTION

The tobacco industry rapidly adapts to new or impending regulations by introducing products with novel design characteristics that exploit regulatory loopholes. To achieve this, the industry frequently uses chemical strategies, replacing regulated chemical product additives with unregulated modified versions (1). This strategy aims to preserve or enhance product addictiveness and appeal while evading regulatory statutes that target the original chemicals. For example, following bans of menthol cigarettes in US federal states such as California and Massachusetts, tobacco companies introduced “non-menthol” cigarettes in which menthol was replaced by an unregulated menthol analogue, WS-3, that elicits similar sensory cooling effects (2, 3). This strategy significantly blunted the expected decline in smoking rates associated with the ban of menthol cigarettes (4, 5).

Industry actors are not just modifying flavour and sensory chemicals but have begun to target nicotine for chemical modification. In 2023, we described a new line of electronic cigarettes introduced in the United States that contains a synthetic nicotine analogue, 6-methyl nicotine (6). These products were introduced to evade federal state bans of flavoured e-cigarettes, bypassing legislation that narrowly defined tobacco products as containing the nicotine molecule but not modified versions of nicotine (6, 7). Products containing 6-methyl nicotine, including additional e-cigarette products and oral pouches, have subsequently become available in many parts of the world (8-11). 6-methyl nicotine (6MN), a synthetic nicotine analogue with higher potency and unknown abuse liability and toxicity, and other nicotine analogues are currently not regulated by the US Food and Drug Administration (FDA) (2, 12-14). The European Union and several US states regulate these compounds as equivalent to nicotine under their tobacco regulatory authority (7, 15). Other jurisdictions, such as Australia and the US state of Indiana, have banned nicotine analogues (16, 17).

Recently, we presented evidence that industry actors in the United States introduced “tobacco-free” combustible cigarettes, named Choice^™^-cigarettes that may contain 6-methyl nicotine (18). Choice^™^-cigarettes are sold without tobacco taxation at a much lower price than tobacco cigarettes (18). Persons 18 years old and above are permitted to purchase the products, at a younger age than the minimum legal age of 21 to purchase tobacco and nicotine in the United States (18). The products are marketed as “nicotine-free”, containing a non-nicotine compound instead, named Novara^™^, promising the same experience as smoking tobacco cigarettes (18). Based on materials available on the vendor website, this non-nicotine compound may be 6-methyl nicotine, however, this has not been independently verified (18).

The vendor also claims that these cigarettes contain non-tobacco “natural plant derivatives”. Since many non-tobacco, or “herbal”, cigarettes contain plant materials sourced from hemp, it is possible that Choice^™^ cigarettes, in addition to 6-methyl nicotine, contain hemp-derived cannabinoids such as cannabidiol (CBD) (19). This study aimed to i) validate the presence of 6-methyl nicotine and cannabinoids, and the absence of nicotine, in Choice^™^ “non-tobacco” cigarettes by chemical analysis, and ii) determine the regulatory challenges posed by these products.

## METHODS

### Sample Selection

Choice^™^-cigarettes were purchased between June and August 2026 from convenience stores in New York City, NY, USA and South Carolina, USA, and the brand online store (bestchoicesmokes.com) in regular and menthol varieties. R. J. Reynolds’ Newport non-menthol (Newport Red) and menthol (Newport Green) cigarettes were purchased at convenience stores in North Carolina, USA, as references, based on their high popularity among people who smoke in the United States.

### Chemicals

Chemicals used for extraction and as reference materials were ethyl acetate (HPLC grade, ≥ 99.7% purity; Millipore Sigma), methanol (HPLC grade, J.T.Baker Chemicals, Avantor Performance Materials); nicotine (tobacco-derived, Millipore Sigma; >99%), 6-methyl nicotine (Toronto Research Chemicals, ~ 97%), L-menthol (Acros Organics, 99%) and Cannabidiol (CBD, Cayman Chemical; 99%), Δ9-THC, Δ8-THC, THC-COOH and deuterated cannabinoids (d3-CBD, d3-Δ8-THC; d3-Δ9 THC, d3-THC-COOH) (Millipore Sigma).

### Extraction of 6-methyl nicotine, nicotine, menthol and cannabinoids

For 6-MN and nicotine, cigarette rod filler and wrapping paper were extracted in 50 mL conical tubes containing ceramic homogenizers (Thermo Scientific, Catalog No. 01-184-141) using a liquid-liquid extraction (LLE) method following established protocols. First, 5N NaOH (150 μL) was added to the tube and vortexed. After incubation for 5–10 minutes, 10 mL of ultrapure deionized water was added and the tubes shaken at 100 rpm for 10 minutes. Next, 10 mL of ethyl acetate were added and shaken for 10 minutes, followed by water bath sonication for 20 minutes. Samples were allowed to stand for 1 hour and then centrifuged at 3600 rpm for 4 minutes. The top organic phase was collected and filtered using a PVDF syringe filter (0.22 μm, CellProTM, Alkali Scientific) for analysis. For cannabinoids, materials were extracted in 15 mL of ethyl acetate in 40 mL glass vials shaken for 2h (@120 rpm), followed by 20 minutes sonication at 70°C and 1h of sedimentation, followed by collection and filtration of aliquots.. 500 μL of the filtrates were air-dried and reconstituted in 500 μL of 90% methanol/water before analysis. For menthol, materials were extracted similar to cannabinoids but using methanol and without the sonication step.

### Analysis of 6-MN, nicotine and cannabinoids by ultra performance liquid chromatography-mass spectrometry (UPLC-MS)

2 μl of each sample were injected into a Waters Acquity I-class plus UPLC with a Waters Acquity UPLC BEH C18 column with mobile phase A (0.1% formic acid in water) and mobile phase B (0.1% formic acid in acetonitrile) at 50°C column temperature. Multiple Reaction Monitoring (MRM) mass spectra were acquired to measure the target signals (6-methylnicotine: m/z 177.0 → m/z 77.0; Nicotine: m/z 163.0 → m/z 117.0, see supplementary table 1 for cannabinoids) with Sciex Triple 7500 + mass spectrometer (Framingham, MA) under positive mode electrospray ionization, with ion spray voltage of 1500 V and source temperature of 400°C for 6-MN and nicotine, and ion spray voltage of 4500 V and source temperature of 500°C for cannabinoids. The curtain gas, ion source gas 1, and ion source gas 2 were 40, 50, and 70 psi, respectively. Sciex OS 4.0.2.188 software used for data acquisition and data analysis was performed using Skyline software (www.skyline.ms). Limit of quantification (LOQ) for both 6MN and nicotine was 4 ng/mL. A deuterated cannabinoid internal standard mixture (d3-CBD, d3-Δ8-THC; d3-Δ9 THC, d3-THC-COOH) was added and mixed with samples before instrument injection (Table 1).

### Analysis of menthol by gas chromatography-mass spectrometry

Menthol was quantified using a GC-2010 (Shimadzu, Columbia, MD, USA) coupled with a GCMS-QO2010 single-quad mass spectrometer with an EI source, fitted with an Agilent HP-5MS column (length 30m, id 0.25 mm diameter, 0.25 μm film; Sigma-Aldrich). The injector temperature was 280°C, the solvent was methanol, and the injection volume was 1 uL with a split ratio of 5. The carrier gas was He at a column flow rate of 0.96 mL/min. The temperature program was an initial hold at 70°C for 2 min, followed by a ramp a 20°C/min to 320°C, then a final hold for 2 min at 320°C. The interface temperature was 250°C, and the MS was operated at 200°C using a scan range of 50–500 m/z. Quantification was performed using extracted ion chromatograms for the target analytes (162 m/z for nicotine, 138 m/z for menthol), and methyl octanoate was used as the internal standard. Samples were quantified relative to a 5-point calibration curve with a linear range from 10–200 μg/mL.

#### Statistical analysis

Tukey’s multiple comparisons test was performed for ChoiceTM-cigarettes, across the two varieties and purchase sources. Dunnett’s multiple comparisons test was performed to determine differences in menthol amounts between Newport menthol-cigarettes and ChoiceTM-cigarettes across purchase source. GraphPad Prism (11.1.0 version) was used for analyzing this data and statistical significance was determined at P < 0.05. This statistical analysis was not pre-registered, and thereby the results should be considered exploratory.

## RESULTS

### 6-methyl nicotine and nicotine contents

6-methyl nicotine was identified in Choice^™^-cigarettes, at similar quantities in both flavour varieties (Regular: 455 ± 59 μg/rod vs. Menthol: 484 ± 36 μg/rod) and across purchase sources, except for Choice^™^-Regular purchased online (381 ± 13 μg/rod)(Table). Newport-Regular and Newport-Menthol contained nicotine at 9119 ± 633 and 7050 ± 721 μg/rod. Nicotine was also detected in both Choice^™^-cigarette flavour varieties (7.4 ± 1.3 μg/rod).

### Menthol contents

Menthol content in Choice^™^-Menthol purchased in New York and online (3362 ± 510 and 3101 ± 180 μg/rod) was higher than in products purchased in South Carolina (2472 ± 297 μg/rod) and NewportMenthol tobacco cigarettes (2222 ± 13 μg/rod). Small quantities of menthol (198 ± 2 μg/rod) were also detected in the Choice^™^-Regular flavour variety.

### CBD and other cannabinoids

CBD and several other cannabinoids were identified in Choice™-cigarettes. CBD contents in Choice™-Regular (9271 ± 1024 μg/rod) were not significantly different from Choice™-Menthol cigarettes (11292 ± 1629 μg/rod). Smaller quantities of other cannabinoids, Δ9-THC (16.5 ± 2.8 μg/rod) and Δ8-THC (3.9 ± 0.4 μg/rod), were detected in all Choice^™^ extracts. No significant differences in cannabinoid contents were identified for between purchase sources or between cigarette flavour varieties (Table 1).

## DISCUSSION

We report the emergence of “tobacco-free” combustible cigarette products containing a synthetic nicotine analogue, 6-methyl nicotine. While 6-methyl nicotine contents determined in these cigarettes are lower than nicotine contents in tobacco cigarettes, 6-methyl nicotine was found to be more potent than nicotine, likely producing significant pharmacological and reinforcing effects in product users (14, 20). The presence of substantial amounts of CBD, along with other cannabinoids, suggests hemp as the filler material.

In the United States, federal state bans of menthol cigarettes and, subsequently, cigarettes containing synthetic menthol analogues, have led to a significant reduction in cigarette sales (4, 5, 7, 21). Choice^™^-Menthol “non-tobacco” cigarettes are marketed in US federal states such as California that ban menthol cigarettes made with tobacco. According to our analysis, Choice^™^-Menthol cigarettes contain menthol at levels similar to US-marketed menthol tobacco cigarettes, representing a likely alternative for people who prefer menthol cigarettes, with similar sensory cooling effects and the typical mint flavour (2). The small amounts of menthol detected in the Choice^™^-Regular variety (~ 0.033% or > ~ 330 PPM) are near thresholds where sensations of flavor, cooling and soothing can be recognized in tobacco cigarettes (22).

In the United States, cigarette sales are further threatened by a 2025 FDA draft rule that proposes to reduce cigarette nicotine contents to non-addictive levels (23). If implemented, millions who smoke are predicted to switch to other forms of nicotine delivery or stop using nicotine products altogether. Unregulated combustible cigarette products such as Choice^™^ cigarettes, containing a nicotine analogue, pose a significant threat to this effort since people who smoke may switch to Choice^™^ cigarettes instead of e-cigarettes or nicotine pouches that FDA deems less risky to use. The abuse liability, appeal, toxicity and health risks from acute and chronic use of 6-methyl nicotine-containing products remain poorly understood. Products such as Choice^™^ cigarettes, containing 6-methyl nicotine, cannabinoids and nicotine, may pose heightened toxicity risks, due to potential pharmacological interactions and the multitude of toxicants produced during combustion.

The United States Congress urgently needs to enable FDA to regulate nicotine analogues, as recently proposed by the agency (24). Once authorized, FDA needs to act quickly to limit availability of nicotine analogue products until their human abuse liability and toxicological properties are fully investigated. Otherwise, as seen in California where nicotine analogue products are regulated based on flavor presence, such products may pass regulatory review and are made available to consumers (25, 26). An alternative regulatory approach was implemented in the state of Indiana that bans the sales of all nicotine analogue products (16). The presence of small amounts of nicotine detected in Choice^™^ cigarettes in this study further justifies their regulation as tobacco products.

## Supplementary Material

This is a list of supplementary files associated with this preprint. Click to download.

• JabbaetalSupplementaryTable1.docx

## Figures and Tables

**Table 1:** Quantification of *S*-6-methyl nicotine (6MN), *S*-nicotine, menthol, cannabidiol (CBD), Δ9-THC and Δ8-THC in Choice^™^ brand “tobacco-free” cigarettes purchased in New York, South Carolina and online, with Newport menthol-flavored and unflavored tobacco cigarettes as references. Data for each analyte is shown as mean μg (SD) / cigarette rod. BLQ is below level of quantification, <LOD is below level of detection and NA is not analyzed.

Product name, Sample purchase location and date	mean μg (SD) / cigarette rod					
	6MN	Nicotine	Menthol	CBD	Δ9- THC	Δ8- THC
Choice^™^ “tobacco-free” cigarettes (manufacturer: Signature Brands USA)						
Choice™-Regular						
NY, June 26 (n = 3–6)	496.8 (23)	8.8 (1.7)	199 (2.3)	9278 (1127)	14.6 (1)	3.6 (0.5)
SC, July 26 (n = 3–4)	467.9 (55)	6.5 (0.9)	197 (1.5)	10571 (199)	16.78 (0.1)	3.6 (0.4)
Online, Aug 26 (n = 3–4)	380.6 (13)^a^	6.6 (1.0)	BLQ	8897 (875)	14.4 (2.5)	3.7 (0.3)
Choice™-Menthol						
NY, June 26 (n = 3–6)	486.1 (33)	7.4 (0.5)	3362 (510)^b^	11461 (1092)	18.6 (0.3)	4.3 (0.4)
SC, July 26 (n = 2–4)	465.9 (35)	7.0 (0.9)	2472 (297)^a^	10812 (2662)	20.7 (0.6)	4.2 (0.1)
Online, Aug 26 (n = 3–7)	499.9 (41)	7.3 (0.9)	3101 (180)^b^	11406 (1928)	17.7 (2.7)	4.2 (0.5)
Newport tobacco cigarettes (manufacturer: RJ Reynolds)						
Newport-Regular (n = 9)	<LOD	9119 (633)	<LOD	<LOD	<LOD	<LOD
Newport-Menthol (n = 3)	<LOD	7050 (721)	2222 (13)	NA	NA	NA

a Significantly different within **Choice^™^** products (p < 0.05; Tukey’s multiple comparisons test)

b Menthol means in **Choice™-Menthol** are significantly different from reference Newport-Menthol product (p < 0.05; Dunnett’s multiple comparisons test)
